# Association of *CX3CR1* Gene Polymorphisms with Fractalkine, Fractalkine Receptor, and C-Reactive Protein Levels in Patients with Kidney Failure

**DOI:** 10.3390/ijerph18042202

**Published:** 2021-02-23

**Authors:** Łukasz Woźny, Joanna Żywiec, Katarzyna Gosek, Roman Kuźniewicz, Sylwia Górczyńska-Kosiorz, Wanda Trautsolt, Mirosław Śnit, Władysław Grzeszczak

**Affiliations:** Department of Internal Medicine, Diabetology and Nephrology, Faculty of Medical Sciences in Zabrze, Medical University of Silesia, 40-055 Katowice, Poland; klops336@wp.pl (Ł.W.); jzywiec@sum.edu.pl (J.Ż.); kgosek@sum.edu.pl (K.G.); rkuzniewicz@sum.edu.pl (R.K.); skosiorz@sum.edu.pl (S.G.-K.); wtrautsolt@sum.edu.pl (W.T.); msnit@sum.edu.pl (M.Ś.)

**Keywords:** fractalkine, fractalkine receptor, inflammation, end-stage renal disease

## Abstract

Fractalkine (*CX3CL1*) is a chemokine that plays a significant role in inflammation, one of the pathophysiological processes underlying end-stage renal disease (ESRD). Genetic factors are significantly involved in cytokine expression and have been studied as potential risk factors for chronic kidney disease (CKD). **Objectives**: We aimed to elucidate the association of *CX3CR1* gene polymorphisms rs3732378 and rs3732379 with the levels of *CX3CL1*, *CX3CL1* receptor (*CX3CR1*), as well as C-reactive protein (CRP). **Patients and methods**: We enrolled 198 participants, including 106 patients with ESRD and 92 controls. Peripheral blood samples were collected from each patient for genetic (rs3732378 and rs3732379 polymorphisms) and immunoenzymatic (fractalkine, *CX3CR1*, CRP) tests. **Results**: *CX3CR1* and CRP levels were higher in patients with ESRD than in controls (*p* < 0.05). Fractalkine levels were significantly higher in ESRD patients who were homozygous for the G allele of the rs3732378 polymorphism and for the C allele of the rs3732379 polymorphism than in homozygous controls. Moreover, carriers of these alleles among patients with ESRD had significantly higher *CX3CR1* levels than controls. **Conclusions**: The G allele of the rs3732378 polymorphism and the C allele of the rs3732379 polymorphism of the *CX3CR1* gene are associated with higher *CX3CL1* and *CX3CR1* levels. Our study suggests that *CX3CR1* gene polymorphisms could be potentially involved in the pathogenesis of ESRD, but the study needs to be replicated in a larger population with a longitudinal follow-up study. Identification of genetic factors associated with inflammation in ESRD may contribute to the development of targeted gene therapies in the future.

## 1. Introduction

Chronic kidney disease (CKD) is a condition in which there is gradual reduction in the number of nephrons and loss of kidney function over time. According to the Kidney Disease Improving Global Outcomes (KDIGO) [1], CKD is defined as structural or functional abnormalities of kidneys, present for at least three months, with important implications for health. Subclinical inflammation has been reported as one of the etiopathogenetic factors [2]. Disease manifestations include excessive accumulation of uremic toxins, malnutrition, atherosclerosis, and chronic inflammation [3], while cardiovascular diseases have been reported as the most common cause of death in this population [4]. The possible reasons for this include low awareness among clinicians about the need for nephroprotection in the treatment of other diseases, as well as the underestimated role of chronic inflammation as the main atherogenic factor [5].

Proinflammatory cytokines and their derivatives (i.e., chemokines) play a major role in the pathogenesis of inflammatory processes [6]. Fractalkine (also termed C-X3-C motif chemokine ligand 1 (*CX3CL1*)) is a unique chemokine of the CX3C subfamily. The highest expression of fractalkine has been demonstrated in the brain, but its presence in other organs has also been reported, including the heart, kidneys, liver, lungs, and adrenal glands [7,8,9]. Fractalkine exists either as a cell-membrane-bound molecule or as a soluble protein [10].

Similar to some other chemokines, fractalkine participates in the regulation of several processes by forming a complex with its receptor, *CX3CR1*, which is encoded by the *CX3CR1* gene located on chromosome 3q21. The *CX3CR1* receptor has been reported to be present on the surface of monocytes and macrophages, in almost all natural killer cells, T cells (CD4^+^, CD8^+^), neutrophils, and other cell types [11,12]. Fractalkine and its receptor are involved in numerous important processes in the body, including strengthening integrin-mediated monocyte adhesion and promoting osteoblast cell migration [13,14]. Its role in the communication between microglial cells and neurons via autocrine and paracrine pathways in the central nervous system has also been demonstrated [15].

As end-stage renal disease (ESRD) has been associated with a higher incidence of cardiovascular events and inflammation, the potential role of *CX3CR1* polymorphisms, namely rs3732378 and rs3732379, has been suggested. Both *single nucleotide polymorphism* (SNPs) (rs3732378 and rs3732379) are located in the exon regions. Their involvement was previously described in the pathogenesis of atherosclerosis and glomerulopathy, which often lead to ESRD [16]. Therefore, the aim of the present study was to (1) compare serum C-reactive protein (CRP), *CX3CL1*, and *CX3CR1* levels between patients with ESRD and healthy individuals, with a separate analysis by sex and by genotype of the *CX3CR1* gene polymorphisms rs3732378 and rs3732379, and (2) assess differences in the distribution of the genotypes and alleles of the rs3732378 and rs3732379 polymorphisms of the *CX3CR1* gene between patients with ESRD and healthy individuals.

## 2. Patients and Methods

### 2.1. Patients

A total of 198 participants were recruited at the Department of Internal Medicine, Diabetology and Nephrology of the Medical University of Silesia (Katowice, Poland), including 106 cases (46 women and 60 men) with ESRD and 92 healthy controls (63 women and 29 men). The mean (SD) age of patients with ESRD was 65.65 (15.47) years and of controls was 26.1 (5.86) years. Peripheral blood samples were collected from each participant for laboratory analysis, including the measurement of CRP levels and creatinine. Kidney function was assessed in all participants based on the estimated glomerular filtration rate (eGFR), calculated using the Cockroft–Gault formula. Moreover, genetic (DNA isolation) and enzyme-linked tests were performed to determine serum *CX3CL1* and *CX3CR1* levels.

The inclusion criteria for cases were as follows: eGFR category G5 according to the Kidney Disease: Improving Global Outcomes (KDIGO) classification, need for hemodialysis (ESRD at the onset of hemodialysis, i.e., eGFR < 15 mL/min/1.73 m^2^), and informed consent to participate in the study. Patients with cancer, chronic steroid treatment, acute infection, CRP levels suggesting secondary inflammation (≤10 mg/L), as well as a lack of informed consent to participate in the study were excluded.

For the control group, the inclusion criteria were as follows: overall good health, absence of chronic diseases, no medication use (either long term or in the previous month), normal body mass and body mass index, no pregnancy, and informed consent to participate in the study. Individuals with a history of chronic diseases, chronic use of any medications, acute infection, and lack of informed consent to participate in the study were excluded.

The study group and the control group differed significantly in terms of age and biochemical parameters, as shown in Table 1. The large age difference between the study and control groups seems to be a limitation of the study. The difference is dictated by the natural course of chronic kidney disease. Patients with end-stage renal disease (stage G5) have numerous comorbidities, which over the years contribute to impaired excretory function of the kidneys. The test group thus formed consisted mainly of elderly people with multiple diseases. It is also worth pointing out that the condition for inclusion in both groups was the fact that there was no coexisting active inflammation, which allowed for the creation of a potential equivalence of both groups. The current scientific research does not confirm that age itself is an isolated factor increasing the concentration of proinflammatory cytokines and chemokines. However, the creation of a study and a control group with a similar age could provide interesting conclusions and seems to be a limitation of the study.

### 2.2. Methods

All laboratory tests were performed at our local laboratory of molecular biology. Whole venous blood samples of about 20 mL were collected from each participant. The CRP level was measured using standard laboratory methods. Serum *CX3CL1* and *CX3CR1* levels were measured by a sandwich enzyme-linked immunosorbent assay using Human Chemokine C-CX-C-Motif Ligand 1 and Human Chemokine C-CX-C-Motif Receptor 1 kits (BlueGene, Shanghai, China). Absorbance was measured at 450 nm using an Infinite f50 reader (Tecan, Männedorf, Switzerland).

Subsequently, genomic DNA was isolated from leukocytes obtained from frozen whole peripheral blood, collected in 400 μL samples. The isolation process was carried out with the MagCore^®^ Compact system for automated nucleic acid extraction (RBC Bioscience, New Taipei City, Taiwan), with the use of magnetic-bead-based technology. An embedded protocol and ready-to-use reagent cartridges were used. The concentrations and quality of isolated DNA were assessed with a DeNovix spectrophotometer (Thermo Scientific, Waltham, MA, USA). Next, fluorescent-labeled probes were applied to identify the genotypes of *CX3CR1* gene polymorphisms using a ready-made SNP genotyping kit, TaqMan Pre-designed SNP Genotyping Assay (Applied Biosystems, Foster City, CA, USA). After polymerase chain reaction, individual alleles were identified by assessing fluorescence in the tested samples using a 7300 Real-Time PCR System analyzer (Applied Biosystems).

### 2.3. Ethical Approval

The study protocol was approved by the Bioethics Committee of the Medical University of Silesia (No. KNW/0022/KB1/70/I/16). The research was conducted in line with the Declaration of Helsinki. All participants provided written informed consent to take part in the study.

### 2.4. Statistical Analysis

To identify significant differences between groups, the *t*-test was used for variables with normal distribution and the Mann–Whitney test was used for variables without normal distribution. The genotype distribution was compared between groups using the χ^2^ test with Yates correction. A *p*-value of 0.05 or less was considered significant. Statistical analysis was performed using Microsoft Excel 2013 (Microsoft Corporation, Redmond, Washington, United States) and Statistica 13.1 (StatSoft Inc., Tulsa, OK, USA).

## 3. Results

There were no significant differences in the distribution of genotypes for the rs3732378 polymorphism between cases and controls (Table 1). The G allele was predominant in both groups (about 70%), while the A allele was present in about 28% of the study group, which is consistent with the frequency of alleles for the rs3732378 polymorphism in the white population (Table 2). The genotype distribution of the rs3732379 polymorphism showed that CT heterozygotes were more common among controls, but the difference was not significant (Table 2).

Mean (SD) CRP levels in the whole study group were 2.57 (2.63) mg/L. In women, the mean (SD) levels were 2.16 (2.38) mg/L, and in men, they were 3.12 (3.14) mg/L. The CRP level was significantly higher in cases than in controls for both sexes and separately for men and women (Table 3). Mean (SD) *CX3CL1* levels in the whole study group were 0.85 (0.58) ng/L; in women, 0.97 (0.68) ng/L; and in men, 0.73 (0.51). Fractalkine levels did not differ significantly between cases and controls either in general or separately for men and women (Table 3). The mean (SD) *CX3CR1* levels for the whole study group were 0.14 (0.1) ng/L; for women, 0.17 (0.12) ng/L; and for men, 0.15 (0.11) ng/L. The mean *CX3CR1* levels were significantly higher in cases than in controls for both sexes as well as separately for men and women (Table 3).

Subsequently, we assessed differences in cytokine levels between groups according to the genotype of *CX3CR1* gene polymorphisms. Differences in CRP levels between cases and controls by genotype are presented in Table 4. The analysis of fractalkine concentrations revealed significant differences between cases and controls for GG homozygotes of the rs3732378 polymorphism and for CC homozygotes of the rs3732379 polymorphism (Table 5). Interestingly, higher *CX3CL1* levels were noted for TT homozygotes among controls than among cases. Finally, *CX3CR1* levels differed significantly between cases and controls for the carriers of the G allele (in both sexes together and separately in men) as well as for GG homozygotes (in women) of the rs3732378 polymorphism. The *CX3CR1* levels differed significantly among carriers of the G allele of the rs3732378 polymorphism (both GG homozygotes and AG heterozygotes), with higher levels observed in cases than in controls. Interestingly, for the rs3732379 polymorphism, *CX3CR1* levels were significantly higher both for CC homozygotes and for rare homozygous carriers of the TT genotype (Table 6).

## 4. Discussion

The pathogenesis of CKD is complex and involves several simultaneous processes that together impair the excretory, homeostatic, and metabolic functions of the kidneys. One of these processes is persistent subclinical inflammation [17]. Numerous studies have reported that inflammatory cells play a central role in kidney damage and are involved in cardiovascular events [18,19].

Upregulation of numerous cytokines and proinflammatory chemokines is an important component of malnutrition–inflammation–atherosclerosis syndrome, which is the major pathophysiological process underlying ESRD [20].

In a large cross-sectional study, Shlipak et al. [21] found that CRP, fibrinogen, and Interleukin-6 [IL-6] levels were higher in patients with renal dysfunction. Moreover, Muntner et al. [22] reported higher CRP, fibrinogen, and homocysteine levels in individuals with eGFR levels lower than 60 mL/min/1.73 m^2^ as compared with those with eGFR levels higher than 90 mL/min/1.73 m^2^. Subsequently, Knight et al. [23] hypothesized that the relationship between renal dysfunction and coronary events was partly mediated by the inflammatory process. They reported that higher levels of high-sensitivity CRP (hs-CRP), IL-6, and soluble tumor necrosis factor receptors type 1 and 2 as well as lower levels of pyridoxal phosphate (PLP) were significantly associated with a higher risk of coronary events in women with a creatinine clearance of less than 75 mL/min as compared with those with a value of 75 mL/min or higher [23].

Our study showed that CRP and *CX3CR1* are significantly higher in patients with ESRD than in healthy individuals. On the other hand, no differences were observed in fractalkine levels between patients with ESRD and controls. This confirms that CKD and its progression may be indirectly associated with an increased inflammatory response, although the potential role of fractalkine alone seems to be less significant.

In a study of Knight et al. [23], increased cardiovascular risk correlated with higher levels of proinflammatory cytokines only in women. The division of the study and control groups according to sex affected the obtained results, especially in analyses stratified by the glomerular filtration rate. In our study, CRP and *CX3CR1* levels were significantly higher both in women and in men with ESRD as compared with healthy female and male controls. On the other hand, fractalkine levels were similar in patients with ESRD and controls irrespective of sex.

In our study, we also assessed associations between selected cytokines or chemokines and the individual genotypes of the studied polymorphisms. The genetic analyses identified two polymorphisms to have potential clinical significance: rs3732378 and rs3732379. Both polymorphic variants are related to the *CX3CR1* gene [24]. Research conducted so far has focused on the role of these polymorphisms in the context of general cardiovascular risk. In 2001, Moatti et al. [25] studied the association between both polymorphisms and the incidence of acute coronary syndrome (ACS). They reported that for the rs3732378 (*CX3CR1*-T280M) polymorphism, carriers of the A allele (i.e., AA homozygotes and AG heterozygotes) had a significantly lower risk of ACS than carriers of the dominant G allele (TM + MM vs. TT), with an adjusted odds ratio of 0.49 (95% CI = 0.27–0.89; *p* = 0.002). For the other polymorphism, a reduced risk of ACS was observed for the carriers of the T allele (I249), while TT homozygotes and CT heterozygotes showed a lower risk of ACS than CC homozygotes (VI + II vs. VV), with an adjusted odds ratio of 0.43 (95% CI = 0.26–0.72; *p* = 0.001).

In 2016, Yadav et al. [26] conducted a study on patients with kidney failure. They assessed the relationships between *CX3CR1* V249I and T280M polymorphisms, fractalkine, and hs-CRP levels in 123 patients with CKD and 100 healthy controls. Fractalkine levels and serum hs-CRP levels were higher in patients with CKD than in controls (*p* = 0.004 and *p* < 0.0001, respectively). However, no associations were found between any of the genotypes and either fractalkine or hs-CRP levels.

In our study, significant differences in CRP concentrations between cases and controls were noted for each genotype of the rs3732378 polymorphism, which suggests that none of those genotypes is associated with a predisposition to higher CRP concentrations among carriers. A similar relationship was noted for the rs3732379 polymorphism.

Our results showed that fractalkine levels were higher among carriers of the GG genotype of the rs3732378 polymorphism in the group of patients with ESRD compared with controls, while no significant associations were noted for the remaining genotypes. As for the rs3732379 polymorphism, significantly higher fractalkine levels were observed for the dominant CC genotype in patients with ESRD when compared with healthy individuals. On the other hand, in the case of the TT genotype, higher fractalkine levels were observed in controls. However, because of a rather small number of participants with this genotype and a large discrepancy in reported concentrations, this association should be considered as a statistical error. It seems that both polymorphisms predispose to higher concentrations of *CX3CL1* only in individuals with dominant genotypes in the population, that is, GG and CC. However, further research is needed to confirm this hypothesis.

Our study also revealed that AG and GG heterozygotes for the rs3732378 polymorphism among patients with ESRD had higher *CX3CR1* levels than carriers of those genotypes among controls. This finding may suggest a potential role of the G allele in predisposing to higher *CX3CR1* levels. The analysis of the rs3732379 polymorphism also revealed significant results for the dominant CC genotype and, interestingly, for the nondominant TT genotype. However, the latter finding may be due to a small number of TT homozygotes (*n* = 18) and should not be regarded as reliable. Thus, our study suggests a potential effect of *CX3CR1* gene polymorphisms rs3732378 and rs3732379 on the concentrations of fractalkine and its receptor. However, further studies in a larger population of patients are needed to confirm these findings.

Our study had several limitations. First, the size of the study population was limited, particularly the control group. A larger sample size would allow us to generalize our conclusions to a wider population. Second, cases and controls differed considerably in terms of age, which is related to the natural course of CKD. However, studies conducted so far did not report age to be an independent factor affecting the levels of proinflammatory cytokines and chemokines. Moreover, our study groups were homogenous in that they included only individuals without active inflammation. Nonetheless, a study on patients with ESRD and age-matched controls could provide some interesting findings.

In conclusion, CRP and *CX3CR1* levels are higher in patients with ESRD than in healthy individuals, while fractalkine levels do not differ between these populations regardless of sex. The G allele of the rs3732378 polymorphism and the C allele of the rs3732379 polymorphism of the *CX3CR1* gene predispose to higher serum *CX3CL1*, *CX3CR1*, and CRP levels in patients with ESRD. Our study suggests the potential involvement of *CX3CR1* gene polymorphims in ESRD, although further studies are needed to fully elucidate this issue. The identification of genetic factors associated with the severity of inflammation in ESRD may help develop an individualized approach to the diagnosis and treatment of these patients based on targeted gene therapy.

## Figures and Tables

**Table 1:** Comparison of clinical and biochemical characteristics of study and control groups.

Parameter	Patients	Controls	*p*-Value
Age (years)	65.65 +/− 15.47	26.1 +/− 5.86	<0.05
Weight (kg)	71.75 +/−16.59	71.46 +/− 9.76	NS
ALT (U/L)	10.44 +/− 6.97	18.76 +/− 11.19	<0.05
AST (U/L)	15.32 +/− 10.02	20.72 +/− 7.23	<0.05
Total cholesterol (mmol/L)	4.21 +/− 1.2	4.61 +/− 0.86	0.02
Creatinine (µmol/L)	571.44 +/277.67	74.56 +/−13.13	<0.05
Estimated glomerular filtration rate (eGFR) (mL/min/1.73m^2^)	10.44 +/− 5.49	114 +/− 24.78	<0.05
Glucose (mg/dL)	113.8 +/− 61.67	85.81 +/− 8.29	<0.05
Triglycerides (mmol/L)	1.66 +/− 1.01	0.89 +/− 0.37	<0.05

Data are presented as the mean (SD). NS, non-significant. ALT, Alanine transaminase. AST, Aspartate Aminotransferase.

**Table 2:** Genotype distribution of the rs3732378 and rs3732379 polymorphims of the *CX3CR1* gene in patients with end-stage renal disease (cases) and controls.

Genotypes of *CX3CR1* Polymorphisms		Patients (*n* = 106)	Controls (*n* = 92)	*p*-Value
rs3732378	GG	69 (65.09)	57 (61.71)	NS
	AG	31 (29.25)	29 (31.91)	
	AA	6 (5.66)	6 (6.38)	
rs3732379	CC	65 (61.32)	40 (43.98)	NS
	CT	32 (30.18)	43 (46.74)	
	TT	9 (8.5)	9 (9.78)	

Data are presented as the number (percentage) of participants. NS, non-significant.

**Table 3:** Differences in C-reactive protein (CRP), *CX3CL1*, and *CX3CR1* levels between patients with end-stage renal disease and controls in general and separately for women and men.

Parameter	Patients (*n* = 106)	Controls (*n* = 92)	*p*-Value
CRP, mg/L	3.89 (2.78)	1.09 (1.39)	<0.05
CRP in women, mg/L	3.55 (2.58)	1.14 (1.42)	<0.05
CRP in men, mg/L	4.15 (3.48)	0.98 (1.08)	<0.05
*CX3CL1*, ng/L	0.91 (0.65)	0.77 (0.48)	NS
*CX3CL1* in women, ng/L	1.06 (1.13)	0.87 (0.66)	NS
*CX3CL1* in men, ng/L	0.8 (0.54)	0.58 (0.41)	NS
*CX3CR1*, ng/L	0.17 (0.11)	0.11 (0.09)	<0.05
*CX3CR1* in women, ng/L	0.19 (0.13)	0.11 (0.08)	<0.05
*CX3CR1* in men, ng/L	0.16 (0.11)	0.09 (0.08)	<0.05

Data are presented as the mean (SD). NS, non-significant.

**Table 4:** Differences in mean C-reactive protein (CRP) levels between patients with end-stage renal disease (cases) and controls by the genotype of rs3732378 and rs3732379 polymorphisms.

Genotypes of *CX3CR1* Polymorphisms		Mean CRP Levels, mg/L		*p*-Value
		Patients	Controls	
rs3732378	AA	3.73 (2.66)	1.07 (1.37)	<0.05
	AG	3.91 (2.84)	1.12 (1.45)	<0.05
	GG	3.9 (2.82)	1.08 (1.38)	<0.05
rs3732379	CC	3.91 (2.83)	1.13 (1.47)	<0.05
	CT	3.94 (2.86)	1.05 (1.33)	<0.05
	TT	3.5 (2.43)	1.08 (1.37)	<0.05

Data are presented as the mean (SD). NS, non-significant.

**Table 5:** Differences in mean fractalkine (*CX3CL1*) levels between patients with end-stage renal disease (cases) and controls by the genotype of rs3732378 and rs3732379 polymorphisms.

Genotypes of *CX3CR1* Polymorphisms		Mean *CX3CL1* Levels, ng/L		*p*-Value
		Cases	Controls	
rs3732378	AA	0.66 (0.58)	0.59 (0.51)	NS
	AG	0.73 (0.65)	0.83 (0.69)	NS
	GG	1.01 (1.04)	0.76 (0.78)	<0.05
rs3732379	CC	1.03 (1.05)	0.69 (0.74)	<0.05
	CT	0.77 (0.75)	0.8 (0.79)	NS
	TT	0.76 (0.82)	1.07 (1.02)	<0.05

Data are presented as mean (SD). NS, non-significant.

**Table 6:** Differences in the mean fractalkine receptor (*CX3CR1*) levels between patients with end-stage renal disease (cases) and controls by the genotype of rs3732378 and rs3732379 polymorphisms.

Genotypes of *CX3CR1* Polymorphisms		Mean *CX3CR1* Levels, ng/L		*p*-Value
		Cases	Controls	
rs3732378	AA	0.22 (0.19)	0.09 (0.08)	NS
	AG	0.15 (0.12)	0.11 (0.09)	<0.05
	GG	0.18 (0.16)	0.11 (0.09)	<0.05
rs3732379	CC	0.19 (0.17)	0.11 (0.1)	<0.05
	CT	0.15 (0.12)	0.16 (0.14)	NS
	TT	0.21(0.19)	0.1 (0.09)	<0.05

Data are presented as the mean (SD). NS, non-significant.

## Data Availability

Not applicable.
